# Patient-Reported Health-Related Quality of Life Impact and Symptom Severity in Patients with Lyme Borreliosis in the Burden of Lyme Disease (BOLD) Study

**DOI:** 10.3390/pathogens15070701

**Published:** 2026-07-02

**Authors:** Holly Yu, Amanda R. Mercadante, Kate Halsby, Alexandra Loew-Baselli, Ye Tan, Juanita Edwards, Frederick J. Angulo, Elizabeth Begier, Mendwas Dzingina, Johan S. Berglund, Anna Moniuszko-Malinowska, Franc Strle, James H. Stark, on behalf of the BOLD Study Group

**Affiliations:** 1 Pfizer Inc., HTA Value and Evidence, Collegeville, PA 19426, USA; juanita.edwards@pfizer.com (J.E.); frederick.j.angulo@pfizer.com (F.J.A.); 2 Pfizer Inc., HTA Value and Evidence, New York, NY 10001, USA; amandarmerc@gmail.com; 3 Pfizer Research & Development, Tadworth, Surrey KT20 7NS, UK; kate.halsby@pfizer.com (K.H.); mendwas.dzingina@pfizer.com (M.D.); 4 Pfizer Research & Development/Pfizer Corporation Austria GmbH, 1210 Vienna, Austria; alexandra.loew-baselli@pfizer.com; 5 Pfizer Inc., Evidence Generation Statistics, Cambridge, MA 02139, USA; ye.tan@pfizer.com (Y.T.); james.h.stark@pfizer.com (J.H.S.); 6 Pfizer Healthcare Ireland Unlimited Company, The Watermarque Building, Ringsend Road, Dublin 4, D04 K7N3 Dublin, Ireland; elizabeth.begier@pfizer.com; 7 Department of Health, Blekinge Institute of Technology, Valhallavägen 1, 371 79 Karlskrona, Sweden; johan.sanmartin.berglund@bth.se; 8 Department of Infectious Diseases and Neuroinfections, Medical University of Białystok, ul. Zurawia 14, blok D, 15-540 Białystok, Poland; anna.moniuszko-malinowska@umb.edu.pl; 9 Department of Infectious Diseases, University Medical Centre Ljubljana, Japljeva 2, 1525 Ljubljana, Slovenia; franc.strle@kclj.si

**Keywords:** Lyme borreliosis, Lyme disease, localized LB, disseminated LB, patient-reported outcomes, quality of life

## Abstract

Lyme borreliosis (LB) can manifest as a localized infection or, if left untreated, disseminated disease. This analysis used Patient-Reported Outcome tools to assess general health, fatigue, pain, and cognitive function in patients with localized or disseminated LB, compared with age- and site-matched controls without LB. All participants were enrolled in the Burden of Lyme Disease (BOLD) study; LB cases were assessed at 3 time points: the enrollment visit, and two follow-up visits conducted within 10 months of enrollment. Controls were evaluated at 2 time points: the enrollment contact and a follow-up contact 16–18 months post-enrollment. Symptom severity and general health quality between groups were compared using two-tailed Student’s *t*-tests and multivariate regression analyses. Disseminated LB cases reported greater fatigue and pain than localized LB (disseminated LB fatigue scores: 4.1, 3.7, and 3.5 at Visits 1, 2, and 3, respectively vs. localized LB: 2.9, 3.0, and 2.9; disseminated LB pain scores: 2.5, 1.6, and 1.3 vs. localized LB: 0.9, 0.6, and 0.8) and controls (fatigue score: 3.2; pain score: 1.0). Disseminated LB was also associated with reduced health-related quality of life. The results of this study indicate that health-related quality of life may vary by disease stage and that patients with disseminated disease showed persistent impairment during the acute phase and at 10-month follow-up.

## 1. Introduction

Lyme borreliosis (LB) is an infectious disease caused by *Borrelia burgdorferi sensu lato* that is transmitted by ticks. An analysis of surveillance data and epidemiological studies from 2015 to 2023 estimates that approximately 132,000 cases of LB are reported annually in Europe [1]. Early LB manifests with localized infection at the site of the tick bite and an erythema migrans (EM) rash. Additional symptoms such as headache, malaise, and body aches may occur, while fever and swollen lymph nodes are rare (<5% of European patients with EM) [2,3]. Localized disease generally lasts for a few weeks [4,5]. If left untreated, weeks to months later, the initial infection can progress to disseminated disease with skin, heart, nervous system, or joint involvement, which may be associated with mild to severe and even debilitating systemic symptoms [3]. Cardiac involvement (Lyme carditis) can manifest with atrioventricular block or pancarditis [3,4]. Neurological manifestations, known as Lyme neuroborreliosis, can present as meningoradiculoneuritis, meningitis, and motor or sensory disturbances [4,5]. Lyme arthritis is characterized by intermittent or enduring inflammation in one or more large joints, especially of the knee [3]. In addition to single (localized) and multiple EM, other skin conditions caused by LB can include borrelial lymphocytoma [4]. Approximately 80% of European LB cases present with the EM manifestation [5]. In disseminated infection, which often presents following an EM, LB can involve the nervous system or joints, presenting as Lyme neuroborreliosis or Lyme arthritis, or as cutaneous manifestations such as acrodermatitis chronica atrophicans (ACA), or borrelial lymphocytoma; the latter two clinical manifestations are exclusive to tick bites in Europe [5,6].

LB impacts patients’ health-related quality of life (HRQoL), due to potential systemic involvement and symptoms associated with localized and disseminated disease [7]. Even in some patients who have received treatment for LB, complaints of generalized symptoms such as widespread joint and muscle pain, fatigue, and cognitive deficits are noted to continue or recur after the acute illness [3,8]. Some patients may also experience physical impairments that can substantially interfere with their lives and social activities [9]. LB studies have utilized Patient-Reported Outcomes (PRO) tools such as the 36-item Short Form (SF-36) health survey, Cognitive Failure Questionnaire (CFQ), Checklist Individual Strength (CIS), and Fatigue Severity Scale (FSS) to evaluate and compare general health and symptom severity in impacted patients [10,11,12,13,14,15,16]. However, prior evidence on HRQoL and symptom burden among patients with LB has been inconsistent, with some studies reporting persistent fatigue, pain, and cognitive impairment after treatment, while others found no significant differences compared with reference populations [10,13,14,15,17]. These inconsistencies may partly be explained by differences in study populations and clinical manifestations studied, limited representation of disseminated disease, reliance on self-reported outcomes, variable follow-up durations, and incomplete characterization of long-term symptom trajectories beyond one year. Together, these limitations contribute to ongoing uncertainty regarding the magnitude and persistence of symptom burden following LB, underscoring the need for large, well-characterized prospective studies such as the Burden of Lyme Disease (BOLD) study. The BOLD study utilized the aforementioned PRO tools, including the Short Form McGill Pain Questionnaire (SF-MPQ), to capture HRQoL and symptom severity at multiple time points following LB diagnosis. Data from BOLD on the incidence of LB, and on manifestation and symptoms of LB have been published elsewhere [18,19]. This prospective, multi-country study enrolled a large and diverse European population, including patients with localized and disseminated disease, providing a unique opportunity to measure symptom severity, assess impact on HRQoL, and compare outcomes between localized and disseminated disease [18,20].

## 2. Materials and Methods

### 2.1. Study Design and Setting

This study used the dataset from the BOLD study, for which the study design and protocol were published in 2023 [18]. Patients were enrolled in the study between April 2021 and July 2022. Cases of any age presenting with symptoms associated with LB were initially enrolled as suspected LB cases from 14 participating general or primary care practices located in endemic areas of the Czech Republic (3 sites), Germany (5 sites), Poland (2 sites), Slovakia (2 sites), Slovenia (1 site), and Sweden (1 site). Following clinical evaluation and standard of care diagnostic testing where appropriate, a final LB diagnosis was assigned according to established criteria for localized or disseminated disease, as described in the study protocol [18]. Participants enrolled as suspected cases who did not receive a final diagnosis of LB were excluded from the present analysis. While the original BOLD methodology paper described three sites in Poland, for the purposes of this analysis, two were combined, as one site served as a referral center for the other.

### 2.2. Study Participants

This analysis included only medically-attended LB cases, defined as participants who sought care at a participating primary care practice and received a final clinical diagnosis of LB following evaluation. Localized LB (EMs) was diagnosed based on characteristic clinical findings, whereas disseminated LB required both compatible clinical manifestations and laboratory confirmation, in accordance with the BOLD case definition and the study protocol [18]. Participants initially enrolled as suspected LB cases but not confirmed to have LB were excluded from all analyses. A diagnosis of LB was based on clinical observation of characteristic signs and symptoms of LB, with laboratory confirmation (at least one positive diagnostic test result from serum and/or skin biopsy specimens) also required for disseminated manifestations [18]. Clinical LB manifestations were categorized based on the definitions outlined in Table 1, which were modified from consensus case definitions developed by the European Union Concerted Action on Lyme Disease (EUCALB) [21].

LB cases were followed in BOLD for up to three visits over one year: an enrollment visit (Visit 1), a follow-up visit during the acute phase (Visit 2), and a second follow-up visit to assess for post-treatment Lyme disease syndrome (PTLDS; Visit 3). Patients with suspected or diagnosed LB were enrolled at Visit 1 (Day 1). Visit 1 included informed consent/assent (as appropriate), a patient or guardian interview, medical record review, and collection of LB symptoms and risk factors. Study-specific procedures were also performed, including blood sampling for serology, photography of manifestations, and skin punch biopsy when applicable. Patients followed one of two acute phase visit schedules depending on the time since their initial LB diagnosis at enrollment. If Visit 1 occurred ≥21 days after the initial diagnosis, all acute phase data were collected at Visit 1, and no Visit 2 was scheduled. If Visit 1 occurred <21 days after diagnosis, Visit 2 was scheduled approximately 21–35 days after diagnosis to complete acute phase follow-up in confirmed LB cases. Visit 2 included a second blood sample, an updated clinical assessment, and administration of the PRO instruments (SF-36, FSS, and SF-MPQ). All LB cases were invited to return for Visit 3 approximately 10 months after diagnosis, regardless of whether Visit 2 was completed. Visit 3 included a third blood sample, interview, and medical record review, assessment of persistent symptoms and Infectious Disease Society of America (IDSA) PTLDS criteria, collection of Charlson Comorbidity Index (CCI) information, and administration of PRO instruments (SF-36, FSS, SF-MPQ, and CFQ). 

Diagnosed LB cases were age-matched (±5 years) 1:1 with a convenience sample of consenting controls from the same participating sites who did not have active LB in the last 90 days prior to enrollment. For each enrolled participant with a final LB diagnosis, six to ten potentially eligible practice panel patients in the same age group and without current LB were selected at random and were invited to participate as controls as soon as possible after case diagnosis. Controls were randomly selected from the same primary care practice panels as the LB cases to represent the underlying source population served at each site. Controls who later became suspected LB cases were excluded from all PRO analyses and denominators as described previously [18]. Control subjects had up to 2 contacts (e.g., either in-clinic or phone consultations) with a primary healthcare professional from a participating primary care site as described previously [18]. In brief, Contact 1 took place on Day 1 and included consent and/or assent, assignment of participant number, interview or medical record review to collect demographic information, LB risk factors, past tick-borne disease history, and interest in future studies. For controls who reconsented, Contact 2 occurred 16–18 months after the first contact and included an updated medical record review and administration of PRO tools (SF-36, FSS, SF-MPQ, and CFQ). Only one control (from Contact 2) per case was used in the PRO analyses; however, some controls with a second contact were matched to cases who did not return for Visit 3.

### 2.3. Outcomes

Supplemental Table S1 outlines the visit and assessment schedule for the study. Patient demographic characteristics, including age, gender, and country, were collected at Visit 1. Manifestations data were documented at the final diagnosis at Visit 2 (or at Visit 1 for patients who enrolled late and did not have a second visit). At Visit 3, clinical manifestations were reassessed to evaluate whether initial LB symptoms had resolved or stabilized following treatment. Investigators also identified any persistent or new symptoms and determined whether patients met the IDSA criteria for PTLDS. Comorbidity status was determined using the CCI, based on information obtained through the collection of prespecified medical history of clinical significance at Visit 3 (LB cases) and Contact 2 (control subjects) as previously described [18].

At each visit, LB cases (Visits 1, 2, and 3) and controls (Contacts 1 and 2) were evaluated by investigator-led interviews for disease-related symptoms, including fatigue, musculoskeletal pain, and cognitive impairment, which were confirmed through medical records. PRO data on symptom severity and quality of life were collected using validated, standardized questionnaires administered in their original format. HRQoL, severity of fatigue, pain, and cognitive function outcomes were collected from LB cases and the matched controls. HRQoL was measured by the SF-36, which has eight domains scored from 0 to 100 (higher scores indicate better health status): physical functioning, bodily pain, role limitations due to physical and emotional problems, emotional well-being, social functioning, energy/fatigue (vitality), and general health perceptions [22]. The Physical Health Component Summary (PCS) and Mental Health Component Summary (MCS) scores of the SF-36 combine several of the health domains to assess how physical or mental health limitations affect daily activities and an individual’s overall perception of their health. A higher PCS score indicates better physical health and fewer disabilities, while a higher MCS score indicates better mental health [22]. Fatigue was measured using the FSS, which assesses the severity of fatigue and its impact on activity and lifestyle by averaging scores from 1 (“strongly disagree”) to 7 (“strongly agree”) across 9 items [23]. A mean score of ≥4 indicates clinically relevant fatigue, and ≥5 denotes severe fatigue [23]. Pain was measured using the SF-MPQ, which is rated on an intensity scale from 0 (none) to 3 (severe) for 15 descriptors [24]. Higher scores across these components indicate greater pain burden [24]. While no universal cutoffs exist for mild, moderate, or severe pain, interpretation is based on relative score magnitude, and clinically meaningful differences are typically judged by changes in these scores rather than fixed thresholds [24]. Cognitive function was measured by the 25-item CFQ, which assesses the frequency of everyday cognitive lapses using the total sum from a 5-point scale from 0 (never) to 4 (very often), yielding a total score of 0–100 [25]. Higher scores indicate more frequent cognitive failures; although no standardized clinical threshold exists, scores in the upper quartile typically reflect significant impairment [25]. Higher scores on FSS, SF-MPQ, and CFQ indicate more debilitating fatigue, intense pain perception, and frequent cognitive failures, respectively, whereas higher SF-36 scores indicate better health status. These PRO tool measurements provide context for interpreting symptom severity and HRQoL impacts.

LB cases completed the SF-36 and SF-MPQ at either Visit 1 or Visit 2 and then again at Visit 3. The FSS was completed at all visits, and the CFQ was completed at Visit 3. The SF-36, FSS, SF-MPQ, and CFQ were administered at Contact 2 (16–18 months after enrollment) for controls. Measurements from patients who completed the whole questionnaire (answered all items) were included in the analysis. Mean domain and total scores were compared between LB manifestation subgroups and matched controls.

### 2.4. Statistical Analysis

The data were analyzed in SAS version 9.4 (SAS Institute, Cary, NC, USA). Data were summarized with frequency (n), proportions (%), means (standard deviation), or medians (interquartile range). Associated 95% confidence intervals (CIs) for the percentages were calculated using the Clopper-Pearson method. Independent two-tailed *t*-test and a multivariate regression analysis (analysis of covariance [ANCOVA]) model were used to compare the PRO scores between LB cases (by visit and by manifestation) and controls. For the ANCOVA model using Restricted Maximum Likelihood (REML) estimation method, the dependent variable was the HRQoL tool score, and the primary independent variable was having LB (e.g., LB case versus control) or manifestation stage (Localized versus Disseminated). The potential confounding covariates adjusted for in the multivariate regression analysis were baseline age, sex, comorbidities (i.e., CCI score), history of LB, and country. A *p*-value of *p* < 0.05 was used to determine the observed significant differences between subgroups.

## 3. Results

### 3.1. Study Population Characteristics

At Visit 1, 315 medically attended LB cases were enrolled; 272 of these attended Visit 2, and 253 attended Visit 3. Analyses were conducted using all LB cases who completed each respective visit (N = 315 at Visit 1, N = 272 at Visit 2, and N = 253 at Visit 3), along with 288 controls. Baseline characteristics for the LB and control groups are shown in Table 2. Across all visits, enrolled participants included slightly more females than males (57% for the LB group and 53% for the control group), and over half fell into the 35–64-year age group (mean age was 56 years for both the LB and the control groups). All LB patients enrolled were non-Hispanic White, and 99.7% of the control group were non-Hispanic White, except for one subject (0.3%) who identified as Asian. The comorbidity level was comparable, with mean CCI scores of 3 in both groups (2.8 for LB cases and 2.5 for controls). About two-thirds (66.7%) of the 315 LB cases were enrolled at Visit 1 with localized disease (EM), and the remainder had Lyme neuroborreliosis (0.6%), Lyme arthritis (25.4%), Lyme carditis (0.3%), other manifestations (4.1%; LB diagnosis without established LB clinical manifestation as defined in Table 1, e.g., systemic symptoms only), or more than one manifestation (2.9%; 6 cases had EM and Lyme arthritis, 1 case had EM and Lyme carditis, 1 case had EM and Lyme neuroborreliosis, and 1 case had Lyme arthritis and ‘other’ manifestation). No patients presented with borrelial lymphocytoma, ACA, or ocular manifestations. Patients comprising the disseminated manifestations group were clinically heterogeneous, with the majority of cases classified as Lyme arthritis (87/105 at Visit 1, 82/95 at Visit 2, and 79/92 at Visit 3). Other disseminated manifestations were less common (data not shown).

### 3.2. Comparison of Mean PRO Scores

**Figure 1:**
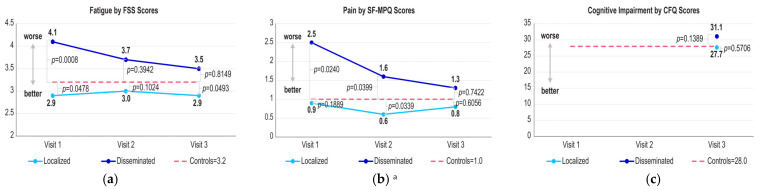
Mean symptom severity scores by manifestation and by visit vs. controls. (**a**) Fatigue by FSS scores (**b**) Pain by SF-MPQ scores; (**c**) Cognitive impairment by CFQ scores. Note: *p*-value versus control shown using multivariate regression analysis. ^a^ Ns for SF-MPQ at Visit 1 are small and represent a different subset of subjects from Visit 2. Abbreviations: CFQ = Cognitive Failures Questionnaire; FSS = Fatigue Severity Scale; LB = Lyme borreliosis; SF-36 = 36-item Short Form Health Survey; SF-MPQ = Short-form McGill Pain Questionnaire.

**Figure 2:**
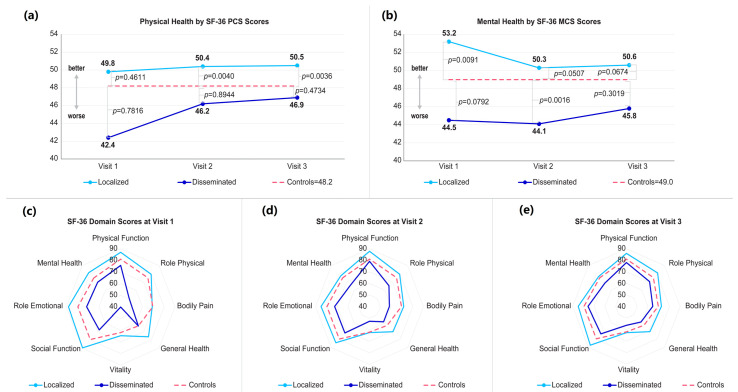
Mean HRQoL scores on the SF-36 ^a^ of LB cases by manifestation and by visit vs. controls. (**a**) Physical Health by SF-36 PCS scores; (**b**) Mental Health by SF-36 MCS scores; (**c**) SF-36 domain scores at Visit 1; (**d**) SF-36 domain scores at Visit 2; (**e**) SF-36 domain scores at Visit 3. Note: *p*-value versus control shown using multivariate regression analysis. ^a^ Ns for SF-36 at Visit 1 are small and represent a different subset of subjects from Visit 2. The PCS and MCS summary scores are standardized to have a mean of 50 based on a normative dataset, while the individual SF-36 domain scores are linearly transformed to a 0–100 scale. Abbreviations: HRQoL = health-related quality of life; LB = Lyme borreliosis; MCS = Mental Health Component Summary; PCS = Physical Health Component Summary; SF-36 = 36-item Short Form Health Survey.

The mean scores across the symptom severity and HRQoL tools and the comparisons between LB manifestation groups and controls across visits are shown in Figure 1 and Figure 2.

#### 3.2.1. FSS

Medically-attended LB cases reported numerically worse fatigue (higher FSS) than controls at baseline (3.3 vs. 3.2), but the mean FSS scores improved from Visit 1 to Visit 3; by Visit 3, the FSS score of LB cases (3.1) was numerically better, but not statistically significant compared to that of controls (Supplemental Table S2). When assessed by manifestation, disseminated LB cases consistently recorded higher (more severe fatigue) FSS scores than both controls and localized LB across all visits, though the mean FSS scores for the disseminated LB subgroup showed improvement over time (Figure 1). Disseminated LB resulted in significantly higher (worse) fatigue scores at Visit 1 (*p* = 0.0008) and showed numerically worse, but not statistically significant, fatigue at both Visit 2 (*p* = 0.3942) and Visit 3 (*p* = 0.8149). Between the two manifestations, the mean FSS scores were significantly higher for the disseminated LB patients compared with EM patients at all three visits, based on multivariate analyses (Visit 1: 4.1 vs. 2.9, *p* < 0.0001; Visit 2: 3.7 vs. 3.0, *p* = 0.0011; Visit 3: 3.5 vs. 2.9, *p* = 0.0077).

Localized LB cases presented with improved FSS scores in comparison to controls. At all visits, the scores on the FSS for localized LB were lower than those for controls, a difference that was statistically significant at Visit 1 (2.9 vs. 3.2, *p* = 0.0478) and at Visit 3 (2.9 vs. 3.2, *p* = 0.0493).

#### 3.2.2. SF-MPQ

As with the case-reported FSS scores, the SF-MPQ scores were higher in the disseminated LB cases than in controls at baseline and improved over time; for pain, scores at Visit 1 were 2.5 vs. 1.0 (*p* = 0.0240) for disseminated LB cases vs. controls at baseline. However, SF-MPQ scores did improve over time (Visit 2 [1.6 vs. 1.0, *p* = 0.0339]) and both LB cases and controls reported similar scores at Visit 3, 1.3 vs. 1.0 (*p* = 0.7422), respectively (Supplemental Table S2). 

Localized LB cases scored better on the SF-MPQ, whereas disseminated LB had worse SF-MPQ scores, when compared to the matched controls (1.0) at all time points (Figure 1). Mean scores were significantly different (worse) than controls for patients with disseminated LB at Visit 1 (2.5, *p* = 0.0240) and at Visit 2 (1.6, *p* = 0.0399), but not at Visit 3. Additionally, disseminated LB had SF-MPQ scores that were worse (indicating more severe pain) than localized LB at all time points; the comparison was significantly different among patients at Visit 2 (localized: 0.6 vs. disseminated: 1.6, *p* = 0.0005) (Figure 1, Supplemental Table S2).

#### 3.2.3. CFQ

The mean CFQ scores were assessed at a single time point (Visit 3). Mean scores did not differ significantly between controls (28.0) and localized LB patients (27.7, *p* = 0.5706), nor between controls and disseminated LB patients (31.1, *p* = 0.1389). Although disseminated LB patients had numerically higher CFQ scores than localized LB patients, this difference was not statistically significant (Figure 1, Supplemental Table S2).

#### 3.2.4. SF-36

As shown in Figure 2, measurements of HRQoL related to physical function, as indicated by the SF-36 PCS score, revealed poorer physical health and greater disability among disseminated LB cases compared to controls, although the results were not statistically significant. Localized LB cases had higher PCS mean scores than controls at all time points. These results showed that patients with EM manifestation had significantly better physical health than matched controls at Visit 2 (localized: 50.4 vs. controls: 48.2, *p* = 0.0040 by multivariate regression model) and at Visit 3 (localized: 50.5 vs. controls: 48.2, *p* = 0.0036 by multivariate regression model). In addition, the PCS mean scores were significantly different between the manifestation subgroups according to the multivariate regression model at Visit 2 (localized: 50.4 vs. disseminated: 46.2, *p* = 0.0012) and at Visit 3 (localized: 50.5 vs. disseminated: 46.9, *p* = 0.0011) (Figure 2, Supplemental Table S2).

The SF-36 MCS mean scores (Figure 2) also indicated that disseminated LB cases reported lower mental health than matched controls; only Visit 2 showed significantly worse mental health (disseminated: 44.1 vs. controls: 49.0, *p* = 0.0016 by multivariate regression model). Compared to the control group, the MCS mean scores among the localized LB patients were significantly higher (indicating better mental health) at Visit 1 (localized: 53.2 vs. controls: 49.0, *p* = 0.0091) and then comparable but numerically higher at subsequent visits. Significant differences in MCS scores were observed between the manifestation subgroups at earlier visits (*p* = 0.0383 at Visit 1 and *p* = 0.0005 at Visit 2).

Compared to matched controls, localized LB cases reported better scores in all SF-36 domains (except for bodily pain at baseline), and conversely, disseminated LB cases demonstrated worse scores across all domains (except for general health at baseline), indicating that a progression to disseminated disease negatively impacts overall HRQoL (Figure 2). Disseminated LB notably showed the greatest HRQoL impact on the vitality domain (Figure 2), which stayed consistently worse than the control group across all visits (Supplemental Table S2). For the localized LB group, scores on the physical function, role physical, general health, social function, and role-emotional domains remained significantly better than controls at all or most time points (Supplemental Table S2). 

Despite SF-36 domain scores from all disseminated LB cases improving over time, disseminated LB cases consistently demonstrated worse scores in all SF-36 domains compared to localized disease (Figure 2). Statistically significant differences between localized and disseminated LB were found in the social function subscale at all visits (disseminated at Visit 1: 67.9 vs. localized at Visit 1: 89.7, *p* = 0.0095; disseminated at Visit 2: 72.2 vs. localized at Visit 2: 84.1, *p* = 0.0013; disseminated at Visit 3: 73.6 vs. localized at Visit 3: 87.5, *p* = 0.0013) and in most of the domains across Visits 2–3 including the physical function domain (disseminated at Visit 2: 79.1 vs. localized at Visit 2: 87.8, *p* < 0.0001; disseminated at Visit 3: 78.0 vs. localized at Visit 3: 86.0, *p* = 0.0028) and the bodily pain subscale (disseminated at Visit 2: 58.4 vs. localized at Visit 2: 71.7, *p* = 0.0005; disseminated at Visit 3: 64.5 vs. localized at Visit 3: 72.5, *p* = 0.0110).

## 4. Discussion

We identified a notable impact on quality of life by LB, particularly disseminated LB. By enrolling a large and diverse patient population spanning six European countries, the BOLD study reports data on the real-world patient-reported burden of LB and its impact during the acute phase and beyond. The study used PRO tools to assess general health, fatigue, pain, and cognitive function in patients with localized or disseminated LB. The HRQoL impact observed at Visit 1 among patients with diagnosed and treated LB may persist through 10 months after diagnosis (Visit 2 and 3) for some patients, particularly those with disseminated diseases. This suggests that the effects of infection do not fully resolve for all patients following antibiotic treatment. Prior to this study, evidence on HRQoL and symptom burden among patients with LB has been mixed [26,27,28,29]. One large study reported more severe fatigue, pain, and cognitive impairment in patients with LB compared with the reference groups at 12 months follow-up after treatment [10], whereas other studies found no significant differences in HRQoL or symptom burden between those with LB and those without LB [13,14,15,17].

In subgroup analyses, patients with disseminated disease reported lower HRQoL compared with matched controls. PRO scores showed poorer outcomes across all questionnaires at all assessed time points. Most patients with disseminated manifestations had Lyme arthritis rather than other forms of disseminated disease. Subgroup analyses indicated patients with Lyme arthritis had numerically worse mean SF-36 (bodily pain and physical function) and CFQ scores relative to those with other disseminated manifestations (data not shown). Notably, however, both Lyme arthritis and other disseminated manifestations experienced worse FSS scores, poorer SF-36 bodily pain and physical function than localized LB patients during the acute phase (Visit 1 and 2), whereas patients with Lyme arthritis continued to report bodily pain and physical impairment at Visit 3 (data not shown). Both groups showed similar levels of fatigue across 3 visits (data not shown).

Mean PRO scores for the disseminated group, however, showed some improvement over the 10 months from diagnosis, approaching levels closer to the scores for the control and localized LB groups, a trend also seen in the European LymeProspect study for FSS, CFQ, and SF-36 bodily pain subscale scores [10], as well as another publication that reported similar HRQoL scores (based on mean SF-36 physical and mental health component scores) between disseminated and EM cases with longer follow-up time [30]. Fatigue, as scored on the FSS, affected quality of life for localized and disseminated LB patients as early as Day 1 of Visit 1, with significantly worse scores compared with matched controls at Visit 1. At all visits, FSS scores were statistically significantly different between disseminated and localized manifestations. Statistically significant differences in SF-MPQ scores between disseminated cases and controls and between localized cases and controls were noted among patients at Visit 1 as well as at Visit 2 (day 28) for disseminated versus control and disseminated versus localized LB patients. The fact that most disseminated LB patients enrolled in the BOLD study had Lyme arthritis could explain the observed SF-MPQ scores suggestive of more severe pain for disseminated LB compared with localized LB. HRQoL has been previously shown to be significantly worsened for patients with Lyme arthritis in terms of their reported pain, physical activity, and mental health impairment based on the SF-36 questionnaire [16]. Indeed, our analysis reported greater disability and poorer mental health among patients with disseminated LB (according to SF-36 PCS and MCS scores) than the localized LB and control subjects. However, it should be noted that the majority of Lyme arthritis cases originated from a single country, and some patients classified as having Lyme arthritis may not have met strict clinical criteria (e.g., absence of swollen joints). Furthermore, patients with confirmed Lyme arthritis typically experience local symptoms rather than systemic manifestations, which differ from the symptom profile observed in our disseminated LB group. These factors may have influenced the pain severity findings and should be considered a limitation of the present study. The CFQ was the only PRO tool that found no significant difference between cases and controls, though only results from a single time point (month 10) were available. The LymeProspect study found significant differences in cognitive impairment between EM patients (N = 1076) and the reference cohorts (N = 4000 in the general population cohort and N = 2405 in the tick bite without clinical LB cohort) at baseline and 12-months later [10]. Taken together, while these findings as a whole provide useful context, our approach highlights differences in disease manifestation and PROs in a broader, more heterogeneous population drawn from six European countries with high LB endemicity.

Our study found that participants with EMs scored as well as, or in some cases better than, controls across PRO assessments of HRQoL and symptom severity, including during the acute phase. While prior longitudinal studies have shown that most LB patients experience improvement over time and return to population norms [10,30], the favorable scores observed among localized cases relative to controls were unexpected. As this study was conducted in primary care settings and limited to medically-attended cases, individuals with mild, transient, or self-resolving symptoms who did not seek medical care were not captured. This may introduce selection bias, whereby the healthiest or most resilient individuals recover and remain engaged in follow-up, while controls drawn from the same routine primary care populations may include individuals with unmeasured chronic health conditions or psychosocial burdens. Differences in healthcare-seeking behavior may also contribute, as diagnosed patients may engage more actively with follow-up care and support, whereas controls may seek care less frequently or avoid reporting symptoms. In addition, some individuals recovering from an acute, well-defined illness may experience changes in self-perceived well-being or coping, potentially influencing PROs. Finally, residual limitations in control selection, including unmeasured differences in socioeconomic status, lifestyle, or mental health, may partly explain the apparent advantage observed among localized cases despite matching on age group. For example, the FSS, SF-MPQ, and CFQ scores were lower (less impaired) for EM cases than for control subjects. Furthermore, EM patients had significantly higher SF-36 scores at most domains and time points, supporting better physical and mental health status than the control group. While these results are in agreement with previous observations in published literature that symptom burden of EM LB was at least comparable to populations without LB [13,14], they differ from the LymeProspect study, which reported more severe fatigue, cognitive impairment, and pain scores in EM patients compared with reference cohorts up to 12 months after treatment. However, important methodological and population differences (cases and non-LB controls) between the two studies should be noted: LymeProspect included patients exclusively from the Netherlands, used CIS for pain assessment, and involved a population with lower healthcare utilization (average one visit), whereas our study examined treated LB patients from six European countries with high endemicity and multiple follow-up visits. These differences may help explain the variation in findings and underscore the value of studying diverse populations and care patterns. It is important to note, however, that EM cases were still impacted by Lyme disease, and higher scores do not imply an absence of disease-related effects. In fact, our study suggests that using each patient as their own control (i.e., comparing their status before and after infection) might provide a more accurate measure of the true impact of Lyme disease in EM cases in future studies. 

The use of PRO tools in this study provides valuable insight into the patient experience during the acute phase and highlights the persistent burden of LB during the follow-up period, especially for those with disseminated LB. These measures offer clinicians and researchers a patient-centered perspective on symptom severity and HRQoL impact, which is critical for understanding the full impact of LB and guiding supportive care. However, PRO tools have inherent limitations; they capture only self-reported symptoms from activated patients who attend follow-up visits. Consequently, the BOLD study may underestimate LB symptoms and HRQoL impacts among patients who did not return for a follow-up visit, and therefore, the findings are likely to reflect a subset of patients with continued healthcare engagement.

## 5. Conclusions

The BOLD study leveraged PRO tools to capture LB patients’ symptom burden and quality of life data across multiple time points in 6 high-endemic European countries. These scores provide insight into patient experiences that may not be evident during brief clinical encounters, offering a more comprehensive understanding of health during and after the acute phase of illness. The results of this study indicate that HRQoL outcomes may vary by disease stage. Patients with disseminated disease showed persistent impairment during acute phase and at 10-month follow-up.

## Figures and Tables

**Table 1:** LB Clinical Case Definitions.

Clinical Presentation	Definition Based on Sign/Symptom	Laboratory Diagnostic(s)
***Localized***		
Erythema migrans ^a^	Characteristic red or bluish-red patch, with or without central clearing	Positive IgG/IgM on serum Bbsl antibody testing OR
Borrelial lymphocytoma	Painless bluish red nodule or plaque, usually on ear lobe, ear helix, nipple, or scrotum	Positive PCR of Bbsl result from biopsy OR Positive culture of Bbsl from biopsy
***Disseminated*** ^b^		
Acrodermatitis chronica atrophicans	Long-standing red or bluish-red lesions, usually on the extensor surfaces of extremities; initial doughy swelling; possible skin induration and fibroid nodules over bony prominences	Positive IgG/IgM on serum Bbsl antibody testing OR Positive PCR of Bbsl result from biopsy OR Positive culture of Bbsl from biopsy
Lyme neuroborreliosis	Meningo-radiculitis (Bannwarth syndrome), facial palsy, meningitis, encephalomyelitis, or cerebral vasculitis	Intrathecal Bbsl IgM and/or IgG antibodies OR Positive intrathecal *Borrelia* antibody index (CSF versus serum) reflecting intrathecal Bbsl antibody production OR Positive PCR of Bbsl result from CSF OR Positive culture of Bbsl from CSF
Lyme carditis	Acute onset of high-degree atrioventricular conduction disturbances, rhythm disturbances, myocarditis, or pancarditis	Positive IgG/IgM on serum Bbsl antibody testing
Lyme arthritis	Marked swelling in one or a few large joints, most often the knee	Positive IgG/IgM on serum Bbsl antibody testing OR Positive PCR of Bbsl result from synovial fluid or tissue OR Positive culture of Bbsl from synovial fluid or tissue
Ocular manifestations	Conjunctivitis, uveitis, papillitis, episcleritis, or keratitis	Positive IgG/IgM on serum Bbsl antibody testing OR Positive PCR of Bbsl result from ocular fluid OR Positive culture Bbsl from ocular fluid

^a^ Multiple EM lesions would be considered a disseminated manifestation. ^b^ Case definitions for disseminated disease included laboratory confirmation [18]. Abbreviations: Bbsl = *Borrelia burgdorferi sensu lato*; CSF = cerebrospinal fluid; EM = erythema migrans; IgG = immunoglobulin G; IgM = immunoglobulin M; LB = Lyme borreliosis; PCR = polymerase chain reaction.

**Table 2:** Demographic and Clinical Characteristics of LB Cases and Control Subjects.

	Enrolled Medically-Attended LB Cases			Controls
	Visit 1 (N = 315)	Visit 2 (N = 272) ^a^	Visit 3 (N = 253)	Contact 2 (N = 288) ^b^
Sex, n (%)				
Male	136 (43.2)	119 (43.8)	110 (43.5)	136 (47.2)
Female	179 (56.8)	153 (56.3)	143 (56.5)	152 (52.8)
Age at consent (Years)				
Mean (SD)	54.5 (16.58)	53.9 (16.94)	55.5 (15.72)	55.8 (15.74)
Range	3–86	3–86	6–86	6–86
n (%)				
0 to 4	1 (0.3)	1 (0.4)	0 (0.0)	0 (0.0)
5 to 17	8 (2.5)	8 (2.9)	5 (2.0)	6 (2.1)
18 to 34	28 (8.9)	25 (9.2)	19 (7.5)	22 (7.6)
35 to 64	178 (56.5)	156 (57.4)	145 (57.3)	164 (56.9)
≥65	100 (31.7)	82 (30.1)	84 (33.2)	96 (33.3)
History of Lyme Disease, n (%)				
Yes	112 (35.6)	91 (33.5)	91 (36.0)	43 (14.9)
No	203 (64.4)	181 (66.5)	162 (64.0)	245 (85.1)
History of known tick bite(s), n (%)				
Yes	190 (60.3)	158 (58.1)	160 (63.2)	176 (61.1)
No	125 (39.7)	114 (41.9)	93 (36.8)	112 (38.9)
Race/Ethnicity, n (%)				
Asian	0 (0.0)	0 (0.0)	0 (0.0)	1 (0.3)
White	315 (100.0)	272 (100.0)	253 (100.0)	287 (99.7)
Not Hispanic or Latino	315 (100.0)	272 (100.0)	253 (100.0)	288 (100.0)
Occupation, n (%)				
No Occupation ^c^	126 (40.0)	107 (39.3)	101 (39.9)	112 (38.9)
Outdoor	50 (15.9)	46 (16.9)	40 (15.8)	39 (13.5)
Indoor	127 (40.3)	109 (40.1)	102 (40.3)	129 (44.8)
Student	7 (2.2)	6 (2.2)	6 (2.4)	6 (2.1)
Missing or Unknown	5 (1.6)	4 (1.5)	4 (1.6)	2 (0.7)
Country, n (%)				
Czechia	32 (10.2)	30 (11.0)	26 (10.3)	31 (10.8)
Germany	42 (13.3)	36 (13.2)	22 (8.7)	24 (8.3)
Poland	9 (2.9)	9 (3.3)	8 (3.2)	7 (2.4)
Slovakia	107 (34.0)	101 (37.1)	96 (37.9)	106 (36.8)
Slovenia	21 (6.7)	21 (7.7)	16 (6.3)	21 (7.3)
Sweden	104 (33.0)	75 (27.6)	85 (33.6)	99 (34.4)
Disease Stage ^d^, n (%)				
Localized	210 (66.7)	177 (65.1)	161 (63.6)	0 (0.0)
Disseminated	105 (33.3)	95 (34.9)	92 (36.4)	0 (0.0)
CCI Score				
n	NA	NA	253	288
Mean (SD)	NA	NA	2.8 (2.10)	2.5 (1.91)

^a^ Not all cases attended Visit 2. For patients who enrolled ≥21 days after LB diagnosis, data collection was performed at Visit 1, and there was no Visit 2. ^b^ These N = 288 controls had Contact 2. Their demographics were collected at Contact 1, and CCI, CFQ, SF-36, and SF-MPQ assessments were collected at Contact 2. ^c^ Persons who do not have an occupation. ^d^ Among the 315 LB cases at Visit 1, 210 had erythema migrans manifestation, 2 had Lyme neuroborreliosis, 80 had Lyme arthritis, 1 had Lyme carditis, 13 had other manifestations, and 9 had more than one manifestation. Abbreviations: CCI = Charlson Comorbidity Index; CFQ = Cognitive Failures Questionnaire; LB = Lyme borreliosis; NA = not applicable; SD = standard deviation; SF-36 = 36-item Short Form Health Survey; SF-MPQ = Short-form McGill Pain Questionnaire.

## Data Availability

The datasets used and/or analysed during the current study are not publicly available due to privacy or ethical restrictions and are available from the corresponding author on reasonable request.

## Supplementary Materials

The following supporting information can be downloaded at https://www.mdpi.com/article/10.3390/pathogens15070701/s1. Table S1: Visit and Assessment Schedule; Table S2: PRO Scores of LB Cases by Manifestation at Visits 1–3 and Controls at Contact 2.

## Abbreviations

The following abbreviations are used in this manuscript:

ACA	Acrodermatitis chronica atrophicans
ANCOVA	Analysis of covariance
BOLD	Burden of Lyme Disease
CCI	Charlson Comorbidity Index
CFQ	Cognitive Failures Questionnaire
CI	Confidence interval
CIS	Checklist Individual Strength
EM	Erythema migrans
EUCALB	European Union Concerted Action on Lyme Disease
FSS	Fatigue Severity Scale
HRQoL	Health-related quality of life
LB	Lyme borreliosis
MCS	Mental Health Component Summary
PCS	Physical Health Component Summary
PRO	Patient-reported outcome
PTLDS	Post-treatment Lyme disease syndrome
REML	Restricted Maximum Likelihood
SF-36	36-Item Short Form Health Survey.
SF-MPQ	Short-form McGill Pain Questionnaire

## Appendix A

Investigators of the BOLD Study group include Dr Lenka Dybova (Doktor Brno S.R.O., Brno, Czech Republic), Dr Miroslav Pavlasek (ADMED, S.R.O., Ceske Budejovice, Czech Republic), Dr Daniela Verdanova (Ordinace praktickeho lekare pro deti a dorost, Jindrichuv Hradec, Czech Republic), Dr Joachim Weimer (Private Practice of Dr. Joachim Weimer, Reinfeld, Germany), Dr Christine Kosch (Private Practice of Dr. Christine Kosch, Pirna, Germany), Dr Kai Mehltretter (Velocity Clinical Research, Leipzig, Germany), Dr. Hans-Detlev Stahl (AmBeNet GmbH, Leipzig, Germany), Dr Viliam Cibik (MUDr. Viliam Cibik, PhD, S.R.O., Pruske, Slovakia), Dr Dagmar Zakova (MUDr. Zakova, S.R.O, Ambulancia vnutorneho lekarstva, Trencin, Slovakia), and Dr Barbara Nieradko-Iwanicka (Lubelskie Centrum Diagnostyczne, Swidnik, Poland and Medical University of Lublin, Lublin, Poland).

## Appendix B

**Table A1:** Ethics committee approval information.

Country	Ethics Committee	Initial Approval Date	Initial Approval Code
Czech Republic	Eticka komise IKEM a FTN Fakultni Thomayerova nemocnice Videnska 800 Praha 4—Krc, 140 59	16 February 2021	12/21+4217/21
Germany	Ethik-Kommission bei der Landesärztekammer Hessen Hanauer Landstrasse 152 Frankfurt am Main, 60314	12 March 2021	2021-2414-zvBO
Germany	Ethikkommission bei der Ärztekammer Schleswig-Holstein Bismarckallee 8–12 Bad Segeberg, 23795	12 March 2021	034/21 m
Germany	Ethikkommission bei der Sächsischen Landesärztekammer Schützenhohe 16 Dresden, SACHSEN 01099	12 March 2021	EK-BR-22/21-1
Poland	Komisja Bioetyczna przy Lubelskiej Izbie Lekarskiej w Lublinie ul. Chmielna 4 Lublin, 20-079	1 February 2021	140/2021/KB/VIII
Poland	Komisja Bioetyczna przy Uniwersytecie Medycznym w Bialymstoku ul. Jana Kilinskiego 1 Bialystok, 15-089	25 March 2021	APK.002.169.2021
Poland	Komisja Bioetyczna przy Okregowej Izbie Lekarskiej z siedziba w Bialymstoku ul. Swietojanska 7 Bialystok, 15-082	21 April 2021	12/2021/VIII
Slovakia	Eticka Komisia Trencianskeho samospravneho Karja K dolnej stanici 7282/20A Trencin, 911 01	10 February 2021	No code provided—the approval references Pfizer’s protocol number: C4601002
Slovenia	Komisija Republike Slovenije za medicinsko etiko Stefanova 5 Ljubljana, 1000	31 May 2021	0120-53/2021-4
Sweden	Etikprövningsmyndigheten Box 2110 Uppsala, 750 02	5 May 2021	2021-00604
