# Supplementary material for: Patient-Reported Health-Related Quality of Life Impact and Symptom Severity in Patients with Lyme Borreliosis in the Burden of Lyme Disease (BOLD) Study
Source: Pathogens. 2026 Jul 2;15(7):701. doi: 10.3390/pathogens15070701 (PMC13414593; doi:10.3390/pathogens15070701)
Supplement: Supplementary file 1 [file pathogens-15-00701-s001.zip › pathogens-4252901-supplementary.pdf]

Supplementary Materials

Supplementary Material S.1

Table S1. Visit and Assessment Schedule.

Assessments	Medically-Attended LB Cases			Controls	
	Visit 1[a] (Day 1)	Visit 2 (Day 28)	Visit 3[b] (10 Month)	Contact 1 (Day 1)	Contact 2 (Month 16-18)
Baseline Demographics	x			x	x
Charlson Comorbidity Index (CCI)			x		x
SF-36[a]	x	x	x		x
FSS	x	x	x		x
CFQ			x		x
SF-MPQ[a]	x	x	x		x

[a] If an LB case was screened during Visit 1 at least 21 days after the LB diagnosis, Visit 2 data were collected during the same session, and no separate Visit 2 was scheduled.

[b] Visit 3 took place approximately 9–10 months after Visit 2.

Abbreviations: CCI = Charlson Comorbidity Index; CFQ = Cognitive Failures Questionnaire; FSS = Fatigue Severity Scale; LB = Lyme borreliosis; SF-36 = 36-item Short Form Health Survey; SF-MPQ = Short-form McGill Pain Questionnaire.

Table S2. PRO Scores of LB Cases by Manifestation at Visits 1-3 and Controls at Contact 2.

	N[a]	Statistic	Visit Comparison	FSS Score	SF-36: Physical Function Subscale Score	SF-36: Role Physical Subscale Score	SF-36: Bodily Pain Subscale Score	SF-36: General Health Subscale Score	SF-36: Vitality Subscale Score	SF-36: Social Function Subscale Score	SF-36: Role Emotional Subscale Score	SF-36: Mental Health Subscale Score	SF-36: Physical Health Component Score (PCS)	SF-36: Mental Health Component Score (MCS)	CFQ Score	SF-MPQ Score
**Visit 1**																
Total LB Cases	315	n[b] (Mean (SD))		313 (3.3 (1.66))	35 (84.7 (18.47))	35 (73.9 (32.24))	35 (65.3 (24.47))	35 (73.6 (21.89))	35 (59.8 (23.79))	35 (85.4 (24.72))	35 (84.8 (24.46))	35 (79.3 (17.62))	35 (48.3 (10.48))	35 (51.4 (12.34))	NA	38 (1.3 (1.70))
Localized	210	n[b] (Mean (SD))		208 (2.9 (1.43))	28 (87.0 (14.16))	28 (79.7 (27.67))	28 (69.1 (24.33))	28 (76.3 (19.98))	28 (64.7 (20.14))	28 (89.7 (17.37))	28 (88.1 (19.83))	28 (81.6 (15.70))	28 (49.8 (8.93))	28 (53.2 (9.91))	NA	30 (0.9 (1.20))
Disseminated	105	n[b] (Mean (SD))		105 (4.1 (1.80))	7 (75.7 (30.20))	7 (50.9 (40.89))	7 (50.4 (20.12))	7 (63.1 (27.52))	7 (40.2 (28.61))	7 (67.9 (40.73))	7 (71.4 (36.91))	7 (70.0 (22.91))	7 (42.4 (14.59))	7 (44.5 (18.74))	NA	8 (2.5 (2.65))
Localized vs Disseminated		p-value	NA	<.0001***	0.3707	0.1185	0.0602	0.2717	0.0666	0.2100	0.2870	0.2429	0.2407	0.2766	NA	0.1321
Localized vs Disseminated		p-value (multivariate regression)	NA	<.0001***	0.3188	0.0887	0.1942	0.0527	0.0502	0.0095***	0.1712	0.0063***	0.2617	0.0383***	NA	0.0545
LB Cases vs Controls		p-value	V1 vs C2	0.2383	0.2801	0.8031	0.3423	0.0092***	0.6090	0.1967	0.2281	0.1848	0.9531	0.2613	NA	0.3354
LB Cases vs Controls		p-value (multivariate regression)	V1 vs C2	0.7953	0.0527	0.8266	0.9653	0.0253***	0.9782	0.0987	0.2529	0.0538	0.5932	0.1351	NA	0.8463
Localized vs Controls		p-value	V1 vs C2	0.0907	0.0536	0.4324	0.9152	0.0021***	0.4920	0.0072***	0.0372***	0.0438***	0.3808	0.0399***	NA	0.8003
Localized vs Controls		p-value (multivariate regression)	V1 vs C2	0.0478***	0.0403***	0.6271	0.5263	0.0333***	0.2935	0.0079***	0.0806	0.0031***	0.4611	0.0091***	NA	0.1889
Disseminated vs Controls		p-value	V1 vs C2	<.0001***	0.6625	0.1655	0.0454***	0.9888	0.0908	0.4773	0.5893	0.5827	0.3357	0.5534	NA	0.1408

Disseminated vs Controls		p-value (multivariate regression)	V1 vs C2	0.0008***	0.7561	0.1090	0.1678	0.3739	0.0279***	0.1433	0.3525	0.1457	0.7816	0.0792	NA	0.0240***
**Visit 2**																
Total LB Cases	272	n[b] (Mean (SD))		268 (3.2 (1.65))	264 (84.6 (18.82))	264 (74.5 (24.80))	264 (66.9 (26.43))	264 (66.3 (20.68))	264 (58.8 (22.25))	264 (79.8 (24.31))	264 (80.3 (24.28))	264 (73.2 (18.48))	264 (48.9 (8.54))	264 (48.1 (10.78))	NA	268 (1.0 (1.39))
Localized	177	n[b] (Mean (SD))		173 (3.0 (1.56))	169 (87.8 (16.47))	169 (79.5 (23.05))	169 (71.7 (26.47))	169 (70.7 (19.91))	169 (62.4 (22.27))	169 (84.1 (23.01))	169 (84.9 (22.06))	169 (77.6 (17.62))	169 (50.4 (7.96))	169 (50.3 (10.50))	NA	174 (0.6 (1.05))
Disseminated	95	n[b] (Mean (SD))		95 (3.7 (1.73))	95 (79.1 (21.37))	95 (65.5 (25.36))	95 (58.4 (24.26))	95 (58.5 (19.80))	95 (52.6 (20.90))	95 (72.2 (24.81))	95 (72.3 (26.03))	95 (65.5 (17.51))	95 (46.2 (8.92))	95 (44.1 (10.14))	NA	94 (1.6 (1.71))
Localized vs Disseminated		p-value	NA	0.0013***	0.0008***	<.0001***	<.0001***	<.0001***	0.0005***	0.0002***	0.0001***	<.0001***	0.0002***	<.0001***	NA	<.0001***
Localized vs Disseminated		p-value (multivariate regression)	NA	0.0011***	<.0001***	0.0002***	0.0005***	0.2109	0.0033***	0.0013***	<.0001***	0.0080***	0.0012***	0.0005***	NA	0.0005***
LB Cases vs Controls		p-value	V2 vs C2	0.7468	0.0446***	0.6966	0.2371	0.0620	0.0763	0.8972	0.6560	0.2300	0.3991	0.3251	NA	0.8105
LB Cases vs Controls		p-value (multivariate regression)	V2 vs C2	0.3757	0.0013***	0.5240	0.7328	0.0016***	0.4577	0.2135	0.1156	0.6366	0.0451***	0.9457	NA	0.5473
Localized vs Controls		p-value	V2 vs C2	0.1768	0.0004***	0.0753	0.4198	0.0001***	0.8535	0.0473***	0.0143***	0.1343	0.0106***	0.1719	NA	0.0024***
Localized vs Controls		p-value (multivariate regression)	V2 vs C2	0.1024	<.0001***	0.0168***	0.1536	0.0010***	0.3278	0.0065***	0.0007***	0.1381	0.0040***	0.0507	NA	0.0339***
Disseminated vs Controls		p-value	V2 vs C2	0.0147***	0.4580	0.0014***	0.0002***	0.0600	0.0002***	0.0132***	0.0196***	<.0001***	0.0645	<.0001***	NA	0.0040***
Disseminated vs Controls		p-value (multivariate regression)	V2 vs C2	0.3942	0.8714	0.0892	0.0050***	0.1998	0.0118***	0.0615	0.0756	0.0007***	0.8944	0.0016***	NA	0.0399***

**Visit 3**																
Total LB Cases	253	n[b] (Mean (SD))		253 (3.1 (1.61))	250 (83.0 (20.67))	250 (77.1 (22.69))	250 (69.6 (25.86))	250 (66.5 (20.80))	250 (60.4 (20.51))	250 (82.4 (21.55))	250 (81.4 (21.88))	250 (73.6 (17.04))	250 (49.2 (8.99))	250 (48.8 (9.68))	246 (29.0 (15.02))	252 (1.0 (1.45))
		p-value	V1 vs V3	0.3356	0.6629	0.6242	0.4063	0.1187	0.9128	0.5566	0.5047	0.1205	0.6787	0.2948	NA	0.4340
		p-value	V2 vs V3	0.5835	0.5957	0.4956	0.5189	0.9358	0.6571	0.4896	0.7679	0.8988	0.8050	0.6639	NA	0.8308
Localized	161	n[b] (Mean (SD))		161 (2.9 (1.45))	158 (86.0 (19.04))	158 (81.0 (21.61))	158 (72.5 (26.25))	158 (70.9 (20.31))	158 (62.7 (20.04))	158 (87.5 (18.82))	158 (84.8 (21.01))	158 (76.6 (15.59))	158 (50.5 (8.89))	158 (50.6 (9.00))	154 (27.7 (14.08))	160 (0.8 (1.32))
		p-value	V1 vs V3	0.5621	0.7506	0.8113	0.4989	0.2027	0.6317	0.5396	0.4209	0.1289	0.6908	0.2015	NA	0.6899
		p-value	V2 vs V3	0.4865	0.3707	0.5538	0.7724	0.9024	0.8693	0.1433	0.9650	0.6016	0.8693	0.8356	NA	0.1374
Disseminated	92	n[b] (Mean (SD))		92 (3.5 (1.82))	92 (78.0 (22.41))	92 (70.4 (23.03))	92 (64.5 (24.49))	92 (59.0 (19.52))	92 (56.3 (20.76))	92 (73.6 (23.18))	92 (75.7 (22.26))	92 (68.4 (18.22))	92 (46.9 (8.76))	92 (45.8 (10.11))	92 (31.1 (16.31))	92 (1.3 (1.61))
		p-value	V1 vs V3	0.0166***	0.8511	0.2572	0.1212	0.7092	0.1916	0.7227	0.7711	0.8642	0.4496	0.8618	NA	0.2326
		p-value	V2 vs V3	0.4385	0.7403	0.1720	0.0922	0.8606	0.2281	0.6895	0.3317	0.2692	0.5738	0.2517	NA	0.3058
Localized vs Disseminated		p-value	NA	0.0065***	0.0046***	0.0004***	0.0155***	<.0001***	0.0168***	<.0001***	0.0019***	0.0004***	0.0021***	0.0003***	0.0949	0.0166***
Localized vs Disseminated		p-value (multivariate regression)	NA	0.0077***	0.0028***	0.0037***	0.0110***	0.0177***	0.0018***	0.0013***	0.2058	0.1103	0.0011***	0.0767	0.5841	0.5620
LB Cases vs Controls		p-value	V3 vs C2	0.4851	0.2809	0.4078	0.9870	0.0480***	0.3439	0.1523	0.3095	0.3218	0.2227	0.8547	0.4445	0.8853
LB Cases vs Controls		p-value (multivariate regression)	V3 vs C2	0.0864	0.0117***	0.0534	0.3098	0.0010***	0.8484	0.0047***	0.0391***	0.8920	0.0111***	0.4262	0.6471	0.4388
Localized vs Controls		p-value	V3 vs C2	0.0308***	0.0154***	0.0149***	0.2647	0.0001***	0.6954	0.0002***	0.0154***	0.3264	0.0117***	0.0866	0.8332	0.2420
Localized vs Controls		p-value (multivariate regression)	V3 vs C2	0.0493***	0.0026***	0.0305***	0.0613	0.0003***	0.2384	<.0001***	0.0186***	0.3203	0.0036***	0.0674	0.5706	0.6056
Disseminated vs Controls		p-value	V3 vs C2	0.1673	0.2723	0.0837	0.0896	0.0953	0.0202***	0.0368***	0.1769	0.0024***	0.2355	0.0098***	0.1023	0.0882
Disseminated vs Controls		p-value (multivariat	V3 vs C2	0.8149	0.7552	0.5956	0.4872	0.1999	0.3854	0.7284	0.8200	0.1090	0.4734	0.3019	0.1389	0.7422

		e regression)													
**Contact**															
Contact 2	288	n[b] (Mean (SD))	287 (3.2 (1.56))	285 (81.0 (23.42))	285 (75.4 (26.14))	285 (69.6 (26.67))	285 (63.0 (20.53))	285 (62.0 (18.92))	285 (79.6 (24.24))	285 (79.4 (24.00))	285 (75.1 (16.75))	285 (48.2 (9.80))	285 (49.0 (9.93))	284 (28.0 (14.47))	287 (1.0 (1.42))

[a] N = frequency of subjects in the specified category

[b] n = number of subjects enrolled in the study within the specified group who completed the full questionnaire (answered all items).

***Significant p-value where $p < 0.05$.

"p-value" was from independent two-tailed T-test; "p-value (multivariate regression)" was from ANCOVA model with covariates Age, Sex, Country, Lyme History, and CCI score.

Abbreviations: C2 = Contact 2; CCI = Charlson Comorbidity Index; CFQ = Cognitive Failures Questionnaire; FSS = Fatigue Severity Scale; LB = Lyme borreliosis; NA = not applicable; PRO = patient-reported outcome; SD = standard deviation; SF-36 = 36-Item Short Form Health Survey; SF-MPQ = Short-Form McGill Pain Questionnaire; V1 = Visit 1; V2 = Visit 2; V3 = Visit 3.
